# Formation of Key Aroma Compounds During 30 Weeks of Ripening in Gouda-Type Cheese Produced from Pasteurized and Raw Milk

**DOI:** 10.1021/acs.jafc.4c01814

**Published:** 2024-05-03

**Authors:** Philipp W. Duensing, Jörg Hinrichs, Peter Schieberle

**Affiliations:** † Former Chair for Food Chemistry, Faculty of Chemistry, Technical University of Munich, Lise-Meitner-Str. 34, D-85354 Freising, Germany; ‡ Department of Soft Matter Science and Dairy Technology, Institute of Food Science and Biotechnology, University of Hohenheim, Garbenstraße 21, D-70599 Stuttgart, Germany

**Keywords:** stable isotope dilution assay, cheese ripening, aroma compound formation, CAMOLA approach, (^13^C_6_)-l-leucine, (^2^H_3_)-2-keto-4-methylpentanoic acid

## Abstract

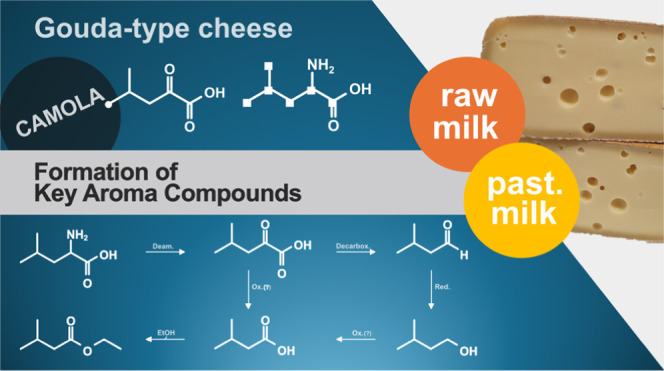

Gouda-type cheeses were produced on a pilot-scale from raw milk (RM-G) and pasteurized milk (PM-G). Sixteen key aroma compounds previously characterized by the sensomics approach were quantitated in the unripened cheeses and at five different ripening stages (4, 7, 11, 19, and 30 weeks) by means of stable isotope dilution assays. Different trends were observed in the formation of the key aroma compounds. Short-chain free fatty acids and ethyl butanoate as well as ethyl hexanoate continuously increased during ripening but to a greater extent in RM-G. Branched-chain fatty acids such as 3-methylbutanoic acid were also continuously formed and reached a 60-fold concentration after 30 weeks, in particular in PM-G. 3-Methylbutanal and butane-2,3-dione reached a maximum concentration after 7 weeks and decreased with longer ripening. Lactones were high in the unripened cheeses and increased only slightly during ripening. Recent results have shown that free amino acids were released during ripening. The aroma compounds 3-methylbutanal, 3-methyl-1-butanol, and 3-methylbutanoic acid are suggested to be formed by microbial enzymes degrading the amino acid l-leucine following the Ehrlich pathway. To gain insight into the quantitative formation of each of the three aroma compounds, the conversion of the labeled precursors (^13^C_6_)-l-leucine and (^2^H_3_)-2-keto-4-methylpentanoic acid into the isotopically labeled aroma compounds was studied. By applying the CAMOLA approach (defined mixture of labeled and unlabeled precursor), l-leucine was confirmed as the only precursor of the three aroma compounds in the cheese with the preferential formation of 3-methylbutanoic acid.

## Introduction

The overall intensity of the aroma of Gouda cheese is known to increase with longer ripening periods. During cheese ripening, the set of microorganisms present initiates a complex enzymatic cascade leading to the partial degradation of mainly lactose, lipids, and proteins via intermediates yielding numerous volatile compounds.^1−5^

Changes in the amounts of cheese volatiles during ripening have been studied in different hard cheese varieties.^6−11^ In particular, increased concentrations of free fatty acids due to lipolysis have been observed,^6,8,9^ and in addition, an increase in some volatiles has been monitored during the ripening of cheese.^7,9−11^ However, in most previous studies, it remained open whether the increase in the volatiles did contribute to the changes in the overall aroma of the respective cheeses. In our recent study, the key aroma compounds of 30 week ripened pilot-scale Gouda-type cheeses produced from pasteurized milk (PM-G) and raw milk (RM-G) were characterized by application of the sensomics approach.^12^

The first aim of this investigation was therefore to investigate the time course of the formation of these aroma compounds during the entire ripening process for up to 30 weeks by means of stable isotope dilution assays. Furthermore, differences in the extent of the formation of single odorants caused by the heat treatment of milk should be elucidated.

With respect to the formation pathways of cheese volatiles, besides lipolysis, the generation of several free amino acids, i.e., the branched-chain amino acids leucine, isoleucine, and valine is considered as important precursors of related cheese volatiles.^4,5,13^ The first model studies establishing the conversion of l-leucine into 3-methylbutanal were performed already 70 years ago.^14,15^ Since then, results of studies on several hard cheeses suggested the microbial formation of 3-methylbutanal, 3-methyl-1-butanol, and 3-methylbutanoic acid from l-leucine or 2-keto-4-methylpentanoic acid.^16−21^ First systematic study using the isolated precursors was performed by Kieronczyk et al.^20,21^ The authors used different microbial strains in model experiments to *in vitro* degrade tritium-labeled l-4,5-(^3^H_2_)-leucine and measured the amounts of the radio-labeled intermediates 2-keto-4-methylpentanoic acid and 2-hydroxy-4-methylpentanoic acid and the radio-labeled volatiles 3-methylbutanal, 3-methyl-1-butanol, and 3-methylbutanoic acid.^20,21^ However, quantitative studies showing the extent of the conversion of l-leucine and 2-keto-4-methylpentanoic acid into the different metabolites using stable isotopically labeled precursors and quantitation with isotopically labeled internal standards in real cheese are not yet available. Therefore, the time course of the formation of 16 key aroma compounds should be followed in RM-G and PM-G cheeses during ripening for up to 30 weeks.

In addition, labeling studies should be performed using the idea of the CAMOLA approach as an isotope enrichment method to measure the correlation between leucine degradation and formation of the three aroma compounds. For this purpose, a laboratory cheese model was developed to monitor the conversion of stable isotopically labeled l-leucine and the labeled intermediate 2-keto-4-methylpentanoic acid into 3-methylbutanoic acid, 3-methylbutanol, and 3-methylbutanal by means of newly developed stable isotope dilution assays.

## Materials and Methods

### Gouda-Type Cheese Made from Raw and Pasteurized Milk

Pilot-scale Gouda-type cheese was produced in cooperation with the Dairy for Research and Training, University of Hohenheim, Germany. Cheeses were produced with either pasteurized milk (PM-G) or raw, nonpasteurized milk (RM-G) as recently described.^12^ Ripening took place under controlled conditions in a climate chamber at 15 °C and 80–85% humidity. Quantitation of aroma compounds was performed in the unripened cheeses (0 week) and after 4, 7, 11, 19, and 30 weeks (batch 1). Cheese production and ripening were repeated after 1 year (batch 2).

### Laboratory Model Cheese for Studies on Precursors

Pasteurized milk (3.5% fat, pH 6.7; 1–2 L) was equilibrated by stirring at 30 °C before a lyophilized starter culture (CHOOZIT Alp D, Danisco Cultures, Copenhagen, Denmark) was added. The precursors (^13^C_6_)-l-leucine and (^2^H_3_)-2-keto-4-methylpentanoic acid were singly added to the milk. During the warm preripening period, a change in pH value was observed. At pH 6.4, lab enzyme was added, and stirring was stopped to allow overnight coagulation. Curd was filled into cheese molds, and the labeled and unlabeled volatiles formed were analyzed after storage for 24 h at 5 °C.

### Chemicals

Reference odorants were obtained from the commercial sources given in parentheses: acetic acid (Merck, Darmstadt, Germany); butanoic acid, δ-decalactone, δ-dodecalactone, ethyl butanoate, ethyl hexanoate, hexanoic acid, 3-methylbutanal, 3-methylbutanoic acid, 3-methyl-1-butanol, pentanoic acid, 2-phenylacetic acid, and 2-phenylethanol (Sigma-Aldrich Chemie, Taufkirchen, Germany).

Diethyl ether, sodium carbonate, sodium chloride, and anhydrous sodium sulfate were purchased from Merck (Germany). Liquid nitrogen was obtained from Linde (Munich, Germany). Dess-Martin periodinane and 4-methylumbelliferyl butanoate were obtained from Sigma-Aldrich Chemie. Diethyl ether was freshly distilled prior to use.

### Isotopically Labeled Internal Standards and Intermediates

The isotopically labeled internal standards used for quantitation of the aroma compounds in the cheeses were synthesized as described recently.^12^

The following differently labeled internal standards needed in the precursor studies were synthesized according to the literature cited: (^2^H_10,11_)-3-methyl-1-butanol^22^ and (^2^H_9_)-3-methylbutanoic acid.^22^ (^2^H_7–9_)-3-Methylbutanal was synthesized from (^2^H_10,11_)-3-methyl-1-butanol by oxidation with Dess-Martin periodinane.^23^

(^2^H_3_)-Acetic acid and (^13^C_2_)-2-phenylacetic acid were purchased from Sigma-Aldrich Chemie, and (^2^H_7_)-2-methylpropanoic acid was from Merck.

(^13^C_6_)-l-Leucine was from Cambridge Isotope Laboratories Inc. (Andover, MA, USA), and (^2^H_3_)-2-Keto-4-methylpentanoic acid was purchased from Sigma-Aldrich (Taufkirchen, Germany).

### Quantitation of the Aroma Compounds in Cheeses and Curd by Stable Isotope Dilution Assays (SIDAs)

Depending on the concentration of the respective odorant determined in preliminary experiments, cheese samples (1–50 g) were powdered with liquid nitrogen. After being spiked with defined amounts of the labeled internal standards, anhydrous sodium sulfate and diethyl ether were added, and the samples were stirred overnight for equilibration. After filtration through defatted cotton wool, the volatile fraction was isolated by SAFE distillation and separated into the acidic and the neutral-basic fraction as recently described.^12,24^ The curd of the laboratory cheeses for precursor studies was worked-up in the same way, but a fractionation of the volatiles into acidic and neutral/basic volatiles was not necessary.

Quantitation was performed by means of a two-dimensional HRGC-MS system (TDGC-MS) using the DB-FFAP capillary column in the first oven and either the DB-FFAP or the DB-5 column in the second oven as previously described.^12^ The MS response factors calculated from the peak areas and the amounts of the labeled and unlabeled compound are summarized in Table S1.

The analysis of the free amino acids and the determination of the lipase activity were performed as previously described.^12^

## Results and Discussion

Based on the identification results obtained in our recent study,^12^ 16 key odorants were quantitated by means of stable isotope dilution assays in both Gouda-type cheeses made from either pasteurized or raw milk. Starting with the unripened cheese taken immediately out of the brine, the formation of aroma compounds was followed at five ripening states for up to 30 weeks. Because the water content of the cheeses decreased during ripening, concentrations were calculated in dry matter (DM). To verify the reproducibility of the cheese production, measurements were carried out in a second batch of the same pilot-scale cheeses after 1 year (batch 2).

### Gouda Cheese Made from Pasteurized Milk (PM-G)

The quantitative results (batch 1) showed increasing concentrations for 12 out of the 16 aroma compounds (Table 1). The highest concentration at all stages was measured for acetic acid; however, acetic acid was already quite high in the unripened curd, and its formation over time was not very pronounced. On the other hand, butanoic acid and 2-methylpropanoic acid showed a strong, continuous increase from 0 to 30 weeks by factors of 16 and 35, respectively, but at different concentration levels. The strongest increase was measured for 3- and 2-methylbutanoic acid, which increased by factors of 60 and 65, respectively. δ-Decalactone and δ-dodecalactone showed already quite high concentrations in the unripened cheese, then slightly increased in the 11 week stored cheese but decreased in the cheese stored for 30 weeks. Butane-2,3-dione and 3-methylbutanal showed a different time course of formation with maximum concentrations after 4 and 7 weeks, respectively, and both were lower in the 30 week stored cheese compared to the unripened cheese. In the same type of cheese, produced after 1 year (batch 2), all trends in odorant concentrations were confirmed (Table S1). Considering the odor thresholds of the aroma compounds, it is obvious that a cheese sold after 6 weeks will show milder aroma attributes, for example, caused by the lactones and butane-2,3-dione, while the odor profile of the older cheeses elicits the more pronounced “cheesy” odor attributes of the short chain fatty acids.

**Table 1:** Concentrations of 16 Key Aroma Compounds in Six Ripening Stages of PM-G Cheese (Batch 1)

		concn (μg/kg DM)^a^					
	compound	after 0 week	4 weeks	7 weeks	11 weeks	19 weeks	30 weeks
**1**	butanoic acid	5345	13915	33365	41633	57362	86811
**2**	hexanoic acid	5279	9782	9523	9390	11943	14931
**3**	pentanoic acid	121	147	252	306	401	572
**4**	ethyl butanoate	16	17	18	21	32	51
**5**	ethyl hexanoate	2	4	5	6	13	23
**6**	δ-dodecalactone	2419	3096	3525	3853	3641	3762
**7**	δ-decalactone	1920	2484	2656	2717	2407	2372
**8**	butane-2,3-dione	2416	3668	3218	2407	2231	1856
**9**	3-methylbutanal	215	223	272	260	157	136
**10**	3-methyl-1-butanol	99	151	158	165	254	251
**11**	3-methylbutanoic acid	622	8012	27703	33055	32644	37548
**12**	2-methylbutanoic acid	90	1254	3606	4038	4888	5836
**13**	acetic acid	712539	934606	1116706	1135269	1066276	1236805
**14**	2-methylpropanoic acid	466	6007	7899	11271	13757	16201
**15**	2-phenylethanol	144	203	244	278	276	318
**16**	2-phenylacetic acid	624	677	859	1221	1302	1899

a Concentrations calculated in dry matter (DM). Mean value of at least three samples. The standard deviation was below 10%.

### Gouda Cheese Made from Raw Milk (RM-G)

As found for PM-G cheese, also in the cheese prepared from raw milk, acetic acid showed the highest concentrations at all ripening states (Table 2). But as in PM-G, the acid was already present in high concentrations in the unripened cheese and increased by only a factor of 3 after 30 weeks. The straight-chain fatty acids butanoic and hexanoic acid showed a continuous increase over time, and within 30 weeks of ripening, their concentrations increased by factors of 13 and 5, respectively. As in PM-G, the most pronounced increases were found for the branched-chain fatty acids 3- and 2-methylbutanoic acid, as well as for 2-methylpropanoic acid. However, the concentrations of 3- and 2-methylbutanoic acid in the 30 week stored cheeses were higher in the cheese from pasteurized milk compared to the raw milk cheese (Tables 1 and 2). On the other hand, after 30 weeks, the straight-chain fatty acids butanoic and hexanoic were lower in the PM-G compared to the RM-G. Ethyl butanoate and ethyl hexanoate showed the steepest increase after 11 weeks and were highest in the 30 week stored sample (Table 2). After 30 weeks of ripening, both esters were clearly higher in the raw milk Gouda cheese (Table 2) than those in the cheese prepared from pasteurized milk (Table 1). δ-Lactones were already present in high amounts in the unripe cheese (Table 2), did not much increase over time, and showed nearly constant concentrations after about 7 weeks. Butane-2,3-dione and 3-methylbutanal showed the same trend as in the cheese from pasteurized milk (Table 1) and passed through a maximum after 4–7 weeks (Table 2). The same changes in concentrations were observed in a second batch (batch 2; Table S2) of the same type of cheese produced 1 year later, confirming the results obtained for the first batch.

**Table 2:** Concentrations of 16 Key Aroma Compounds at Six Ripening Stages in Gouda Cheese from Raw Milk (RM-G) (Batch 1)

	concn (μg/kg DM)^a^					
compound	after 0 week	4 weeks	7 weeks	11weeks	19 weeks	30 weeks
butanoic acid	10471	34024	49815	60489	103823	134164
hexanoic acid	9571	14687	15043	14744	29033	47060
pentanoic acid	179	247	407	481	783	955
ethyl butanoate	39	41	58	73	130	190
ethyl hexanoate	21	32	61	73	144	224
δ-dodecalactone	2186	3146	3676	3760	3792	3644
δ-decalactone	2167	2437	2412	2460	2329	2315
butane-2,3-dione	1192	1957	2306	1592	1316	635
3-methylbutanal	76	73	117	140	107	77
3-methyl-1-butanol	32	71	88	102	157	154
3-methylbutanoic acid	661	7917	9104	15140	24155	25715
2-methylbutanoic acid	67	658	962	1210	2391	3323
acetic acid	416299	690247	973390	1138369	1130505	1322266
2-methylpropanoic acid	478	6670	8056	10348	14825	16898
2-phenylethanol	120	150	157	156	211	231
2-phenylacetic acid	241	384	524	1148	1454	2395

a Concentrations determined in dry matter (DM). Mean value of at least three samples.The standard deviation was below 10%.

The constant increase in the concentrations of straight-chain fatty acids agreed with earlier data by Kanawija et al. in an accelerated ripened Gouda cheese.^8^ The authors found four times more total free fatty acids after 8 months of ripening. Also, Alewijn et al. reported an increase in free fatty acids (C6–C18) in Gouda ripened for 96 weeks.^9^ It is well-known that lipases from microbial origin either from endogenous or exogenous sources do liberate free fatty acids from triglycerides in cheese.^25^ Albenzio et al. determined the lipase activity in Canestrato Pugliese cheese either made from raw or pasteurized milk and reported a distinct increase in the enzyme activity in both cheeses after 1, 28, and 63 days of ripening.^26^

To elucidate the role of lipase in the Gouda cheeses of this study, the activity of the enzyme was determined using 4-methylumbelliferon butanoate as the reference ester. The results (Table 3) showed a constant increase in the lipase activity from unripened cheese to 30 weeks stored cheese. However, at each sampling point, the activity was higher in the RM-G as compared to the PM-G. These results are in good agreement with the higher concentrations of straight-chain free fatty acids in RM-G compared to PM-G cheese (Tables 1 and 2).

**Table 3:** Lipase Activity in Gouda Cheese Made of Pasteurized Milk (PM-G, batch 1) and Raw Milk (RM-G, Batch 1) at 6 Ripening Stages

	lipase activity (U/g) after^a^					
	0 week	4 weeks	7 weeks	11 weeks	19 weeks	30 weeks
PM-G	0.7	0.9	1.0	1.4	1.6	1.9
RM-G	0.8	1.5	1.9	2.8	3.5	3.4

a 1 U is the conversion of 1 nmol 4-methylumbelliferon butanoate per hour. Mean value of at least three samples.

Although an increase in ester concentrations has earlier been reported for different types of cheese, literature studies did not yet report a clear influence of the heat treatment of the milk.^7,9−11^ Ester biosynthesis is assumed to follow a two-step mechanism starting with the generation of the respective free fatty acids, which are subsequently esterified with ethanol.^27^ This mechanism is supported by results of a model experiment showing the formation of ethyl butanoate *in vitro* by lactic acid bacteria.^28^ The preferential formation of both ethyl esters in RM-G cheese (Table 2) is therefore undoubtedly caused by the higher amounts of free butanoic and hexanoic acids present as precursors for ester formation (Tables 1 and 2).

Alewijn et al. have suggested that lactones in cheeses might be formed by a “direct lactonization” of hydroxy fatty acids still bound to the triglyceride rather than by an acid catalyzed formation from released free hydroxy fatty acids.^29^ Because in our experiments, the lactone formation was not in line with the lipase activity (Table 3), the suggested direct lactonization from the intact triglyceride seems probable for lactone formation.

Butane-2,3-dione is suggested to originate from pyruvate metabolism involving lactose and citrate.^30^ The citrate-positive strain *Lactococcus lactisssp. lactis biovar. diacetylactis*, also used in the production of our Gouda, is well-known to generate the buttery smelling diketone. However, obviously this strain is no longer active during longer ripening because the odorant decreased after 11 weeks (Tables 1 and 2).

**Figure 1:**
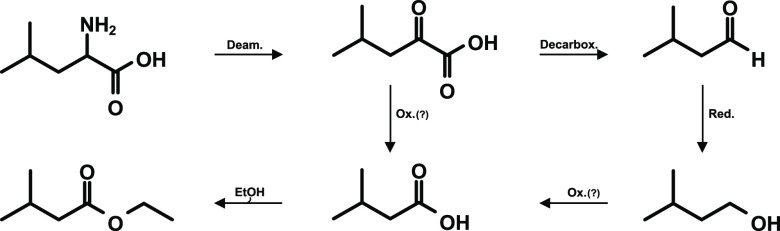
Degradation of l-Leucine in the Ehrlich pathway.

Gouda cheese aroma compounds, such as 3-methylbutanal, 3-methylbutanol, or 3-methylbutanoic acid were earlier reported to be formed by a degradation of the structurally related “parent” amino acid l-leucine.^13^ The formation cascade, known as the Ehrlich pathway,^31,32^ is exemplified for leucine in Figure 1. After deamination into the ketoacid followed by decarboxylation, 3-methylbutanal is formed, which can be reduced to 3-methylbutanol. For the formation of 3-methylbutanoic acid different pathways were discussed in the literature, for example the oxidation of either the alcohol or the aldehyde.^33^ But, also a direct formation of the acid from the intermediate ketoacid by an oxidative decarboxylation may occur.^18^ In agreement with the first steps of the mechanism (Figure 1), 3-methylbutanal in RM-G was formed until week 11 followed by a decrease until week 30. Because the aldehyde is assumed to be reduced to the respective alcohol, consequently, 3-methylbutanol increased after week 11 up to week 30 (Table 1). By contrast, van Leuven et al. had reported a maximum amount of 3-methylbutanol in a Gouda cheese after four months of ripening, followed by a decrease.^11^ In agreement with our data (Tables 1 and 2), Ayad et al.^34^ also reported low amounts of 3-methylbutanal at the end of a ripening period for a Proosdij-type cheese.^35^

The corresponding 3-methylbutanoic acid was by far higher in both Gouda cheeses of this study, indicating a preferential formation of the acid by the Ehrlich mechanism. Besides on l-leucine, the Ehrlich mechanism also applies to the degradation of the amino acids l-isoleucine, l-valine, and l-phenylalanine and the respective acids 2-methylbutanoic acid, 2-methylpropanoic acid, and phenylacetic acid have recently been identified among the key aroma compounds of Gouda cheese.^12^

Because all four acids increased with an increasing ripening time in both types of Gouda cheese (Tables 1 and 2), these data suggested that the concentrations of the four precursor amino acids should also increase with ripening. This assumption was confirmed by the results obtained for PM-G (Table 4) as well as for RM-G (Table 5), showing a clear increase of all four amino acids with an increasing ripening time.

**Table 4:** Concentrations of Selected Amino Acids at Three Ripening Stages of Pasteurized Milk Gouda (PM-G) Cheese (Batch 1)

	concn (μg/kg DM)^a^		
free amino acid	unripened	after 11 weeks	after 30 weeks
l-leucine	867	4446	5927
l-isoleucine	161	1003	2650
l-valine	575	3740	7045
l-phenylalanine	347	1727	2704

a Concentration calculated in dry matter (DM). Mean value of at least three samples. Standard deviation was below 10%.

**Table 5:** Concentrations of Selected Free Amino Acids at Three Ripening Stages of Raw Milk Gouda (RM-G) Cheese (Batch 1)

	concn (μg/kg DM)^a^		
free amino acid	unripened	after 11 weeks	after 30 weeks
l-leucine	524	3181	5212
l-isoleucine	98	669	1923
l-valine	402	2415	5023
l-phenylalanine	272	1384	2317

a Concentration calculated in dry matter. Mean value of at least three samples. Standard deviation was below 10%.

The concentrations of all four amino acids were clearly higher in PM-G (Table 4) compared to RM-G (Table 5). These data are nicely correlated with the higher amounts of, for example, the most pronounced amino acid metabolites 3-methylbutanoic and 2-methylbutanoic acid in PM-G (Tables 1 and 2). Interestingly, ethyl 3-methylbutanoate was not formed from 3-methylbutanoic acid (Figure 1). Although the formation of ethyl butanoate and ethyl hexanoate occurred (Tables 1 and 2), obviously, the set of enzymes present in the cheeses was not able to esterify the methyl branched acids.

### Isotope Labeling Studies on the Formation of Aroma Compounds from Amino Acid Degradation in a Laboratory Cheese Model

**Figure 2:**
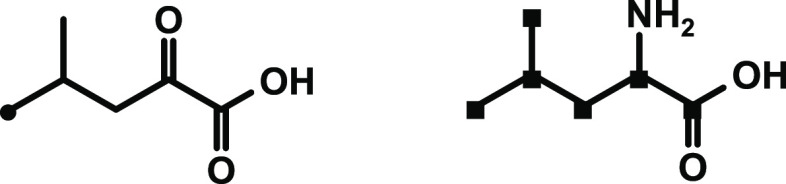
Structures of (^13^C_6_)-l-leucine (black squares show position of carbon-13 label) and (^2^H_3_)-2-keto-4-methylpentanoic acid (black dot shows position of deuterium label) used in the experiments.

The data presented above clearly point to a key role of free amino acids as precursors of several key aroma compounds in Gouda cheese formed by following the Ehrlich mechanism (Figure 1). Thus, to get an insight into the degree of the amino acid degradation in relation to the formation of each single metabolite, either (^13^C_6_)-l-leucine or the intermediate (^2^H_3_)-2-keto-4-methylpentanoic acid (chemical structures in Figure 2) was reacted in a laboratory cheese model. The fat content of the milk, the composition of the starter cultures, the preripening step of the milk, pH, and temperature were identical with the cheese making process. Both isotopically labeled precursors were administered to the milk in single experiments, but it was impossible to equilibrate the precursors in the curd due to its viscous texture. Because the precursors are partly lost with the whey, in a preliminary experiment, the respective amounts of l-leucine were measured before and in the curd and the whey after skimming. In addition, the repeatability of the process was controlled by determination of the water content of the laboratory cheese model each time, resulting in a relative standard deviation of 1.3% (data not shown).

The addition of (^13^C_6_)-l-leucine to a food containing the unlabeled precursor amino acid follows the idea of the CAMOLA approach (carbon module labeling), which was established previously to elucidate reaction pathways and mechanisms in Maillard-type reactions.^36^ A defined mixture of the unlabeled and the completely labeled precursor is reacted to either get mixtures of analyte isotopomers or to show that both the unlabeled and the labeled precursor generate the analytes to the same extent. The amount of the unlabeled precursor, the amino acid leucine, in the curd was, thus, determined in advance. Mass spectrometric data and statistical rules finally allow a conclusion on the importance of the formation pathways of each individual metabolite. In this study, the approach was used to either confirm the amino acid as the only precursor of the three branched-chain l-leucine metabolites or to unequivocally characterize the intermediate ketoacid as the most effective precursor, especially for 3-methylbutanoic acid.

### Method Development for the Quantitation of the Labeled Intermediates by SIDA

During ripening of the cheese model, from the unlabeled, natural l-leucine as well as from the administered labeled leucine, three unlabeled as well as three labeled metabolites are formed in parallel. Because all six intermediates should be quantitated in one run by stable isotope dilution assays, new isotopomers had to be prepared for the quantitation of the labeled (^13^C_5_)-3-methylbutanal, (^13^C_5_)-3-methylbutanol, and (^13^C_5_)-3-methylbutanoic to be expected from the labeled (^13^C_6_)-leucine as well as for the three unlabeled intermediates formed from the natural leucine. The same internal standards were used in the spiking experiment with the (^2^H_3_)-labeled ketoacid for the quantitation of the three labeled (^2^H_3_)-intermediates formed as well as the three unlabeled aroma compounds. The (^2^H_2_)-labeled internal standards recently used in the quantitation of the unlabeled aroma compounds could not be used due to an overlap of some molecular ions in the MS/CI measurements.^12^

**Figure 3:**
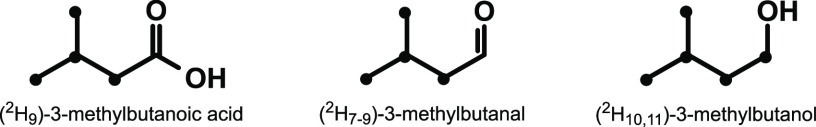
Structures of the labeled internal standards (^2^H_9_)-3-methylbutanoic acid, (^2^H_7-9_)-3-methylbutanal, and (^2^H_10-11_)-3-methyl-1-butanol used in the stable isotope dilution assays (black dots for deuterium label) l.

**Figure 4:**
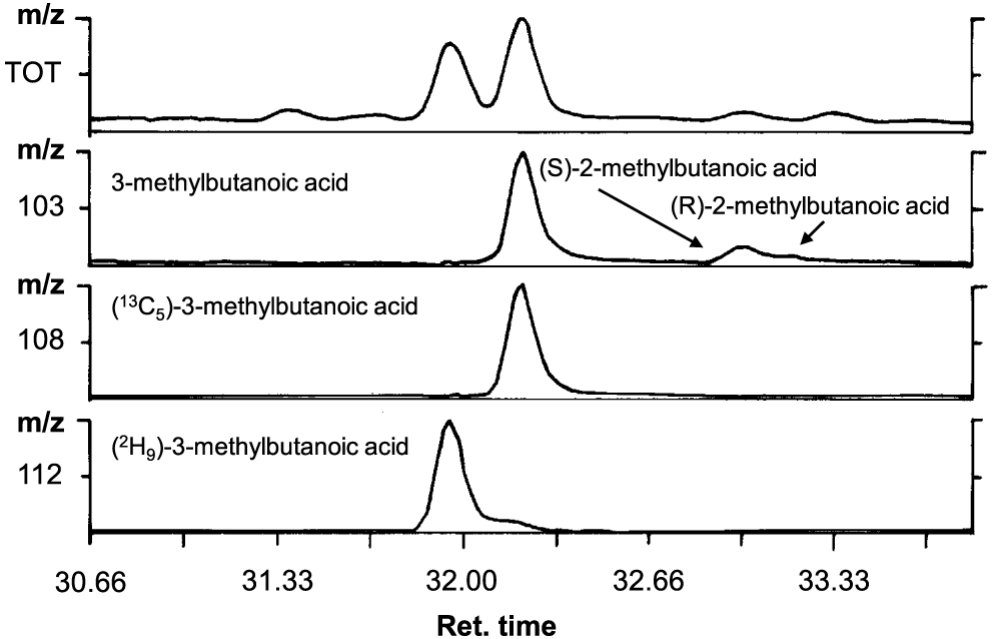
Mass chromatogram of 3-methylbutanoic acid isotopomers in a cheese model spiked with (^13^C_6_)-l-leucine. *m*/*z* 103: 3-methylbutanoic acid and 2-methylbutanoic acid; *m*/*z* 108: (^13^C_5_)-3-methylbutanoic acid; *m*/*z* 112: (^2^H_9_)-3-methylbutanoic acid.

The structures of the new labeled internal standards containing 9–11 deuterium atoms to yield a sufficient mass difference to the analytes are shown in Figure 3. As an example, the mass chromatogram showing the different mass traces for the 3-methylbutanoic acid isotopomers present in a laboratory model cheese sample spiked with (^13^C_6_)-l-leucine is displayed in Figure 4. The mass trace *m*/*z* 103 is generated by MS/CI from unlabeled 3-methylbutanoic acid, which is formed from natural leucine present in the cheese. The mass *m*/*z* 108 represents the degradation product (^13^C_5_)-3-methylbutanoic acid formed from the labeled precursor (^13^C_6_)-l-leucine. Because carbon-13-labeled isotopomers were not separated in the GC stationary phase, both compounds were eluted in one peak. The labeled internal standard (^2^H_9_)-3-methylbutanoic acid is shown in mass trace *m*/*z* 112. Labeling with deuterium usually generates isotopomers that are eluted earlier than the labeled isotopomers. Because for GC separation of the branched fatty acid, a chiral stationary phase was used, the mass trace *m*/*z* 103 not only showed the unlabeled 3-methylbutanoic acid but also the two unlabeled enantiomers of 2-methylbutanoic acid (Figure 4).^36^

### Quantitation of Amino Acid Metabolites in the Laboratory CheeseModel. Conversion of l-Leucine

To get an idea of the extent of the degradation of the amino acid leucine into each metabolite, first, the amount of free leucine was determined in the curd and was calculated on a molar basis. Then, the molar concentrations of the three metabolites 3-methylbutanal, 3-methyl-1-butanol, and 3-methylbutanoic acid formed in the stored laboratory cheese model were determined using the newly developed stable isotope dilution assays. The curd contained an initial concentration of 1124 μmol l-leucine per kg, and in the curd after 24 h storage, concentrations of 0.22 μmol 3-methylbutanal, 0.10 μmol 3-methyl-1-butanol, and 1.61 μmol 3-methylbutanoic acid per kg curd were formed (Table 6). These concentrations corresponded to conversion rates of 0.02% of l-leucine into 3-methylbutanal, 0.01% into 3-methyl-1-butanol, and to a higher extent of 0.15% into 3-methylbutanoic acid. In total, 0.18% of the available free amino acid was converted into the three aroma compounds.

**Table 6:** Volatile Metabolites Formed from l-Leucine Present in a Cheese Model

volatile metabolite	concn (μmol/kg curd)^a^	conversion rate (%)^b^
3-methylbutanal	0.22	0.02
3-methyl-1-butanol	0.10	0.01
3-methylbutanoic acid	1.61	0.15
total	1.93	0.18

a Mean value of at least three samples.

b Initial concentration of l-leucine in the cheese model: 1124 μmol/kg curd.

### Conversion of (^13^C_6_)-l-leucine

Although the free amino acid l-leucine is a precursor of the three aroma compounds, in a biological system such as a curd, other intermediates might have served as precursors for the aroma compounds. To confirm the amino acid as the only precursor, a CAMOLA approach using a defined amount of the stable isotope labeled amino acid in a defined concentration and in the same trial was the method of choice.^35^ The concentration of administered (^13^C_6_)-l-leucine in the cheese model was adjusted to 434 μmol/kg of curd. Well in agreement with the suggested decarboxylation of the amino acid (Figure 4), all three metabolites lost one carbon-13 atom and kept five carbon-13 atoms, as measured by mass spectrometry (Table 7). The conversion rates were determined to be 0.02% for (^13^C_5_)-3-methylbutanal, 0.01% for (^13^C_5_)-3-methyl-1-butanol, and 0.13% for (^13^C_5_)-3-methylbutanoic acid (Table 7). Although, compared to the results for the natural leucine (Table 6), the concentrations of all three metabolites were lower due to the lower concentration of the labeled leucine (Table 6), the conversion rates for all three aroma compounds were nearly identical (Tables 6 and 7). The results established that free leucine is the only precursor of the three aroma compounds with the predominant formation of 3-methylbutanoic acid.

**Table 7:** Amounts of Carbon-13 Labeled Volatile Metabolites in a Cheese Model Spiked with (^13^C_6_)-l-Leucine

volatile metabolite	concn (μmol/kg curd)^a^	conversion rate (%)^b^
(^13^C_5_)-3-methylbutanal	0.07	0.02
(^13^C_5_)-3-methyl-1-butanol	0.04	0.01
(^13^C_5_)-3-methylbutanoic acid	0.57	0.13
total	0.67	0.16

a Mean value of at least three samples. Standard deviation was below 10%.

b Initial concentration of (^13^C_6_)-l-leucine in the cheese model: 434 μmol/kg curd.

The final proof for this suggestion is a comparison of the isotopomeric ratio of the unlabeled/vs the labeled precursor and each unlabeled vs the labeled metabolite.

The ratio in the amino acid is 1124 μmol/kg vs 434 μmol/kg, i.e., a factor of 2.6. Because all three metabolites, within the magnitude of error, showed the same ratio (Table 8), it is confirmed that the formation of the aroma compounds followed the Ehrlich pathway. This way, for example, any new biosynthesis of leucine during the storage of the curd could be ruled out, because this would have affected the ratio of the unlabeled vs the labeled leucine.

**Table 8:** Isotopomeric Ratio in l-Leucine and its Metabolites in a Cheese Model Spiked with (^13^C_6_)-l-Leucine

metabolites	isotopomeric ratio
^12^C_6/_^13^C_6_-l-leucine	2.6
^12^C_5/_^13^C_5_-3-methylbutanal	3.1
^12^C_5/_^13^C_5_-3-methyl-1-butanol	2.9
^12^C_5/_^13^C_5_-3-methylbutanoic acid	2.9

### Conversion of (^2^H_3_)-2-Keto-4-methylpentanoic Acid

2-Keto-4-methylpentanoic acid is suggested as an intermediate in the formation of aroma compounds from the amino acid leucine (Figure 1). To confirm this assumption, (^2^H_3_)-2-keto-4-methylpentanoic acid was administered to the model, and the formation of the labeled metabolites was analyzed. By mass spectrometry, the same deuterium label as that in the precursor acid was found in the methyl group of the three metabolites (Table 9). For (^2^H_3_)-3-methylbutanal and (^2^H_3_)-3-methyl-1-butanol, as in the experiment with the labeled leucine, quite low conversion rates of 0.03% and 0.01% were determined (Table 9). However, from the amounts of (^2^H_3_)-3-methylbutanoic acid formed from (^2^H_3_)-2-keto-4-methylpentanoic acid, a conversion rate of 0.60% was calculated. This conversion rate was five times higher than the formation rate from leucine. This result supports the direct formation of 3-methylbutanoic acid by an oxidative decarboxylation of the 2-keto-4-methylpentanoic acid (Figure 1) rather than the oxidation of the 3-methylbutanol as suggested in other studies.^18,33^

**Table 9:** Deuterium Labeled Metabolites in a Cheese Model Spiked with (^2^H_3_)-2-Keto-4-methylpentanoic acid

metabolite	concn (μmol/kg curd)	conversion rate (%)^a^
(^2^H_3_)-3-methylbutanal	0.11	0.03
(^2^H_3_)-3-methyl-1-butanol	0.05	0.01
(^2^H_3_)-3-methylbutanoic acid	2.27	0.60
total	2.43	0.64

a Initial concentration of (^2^H_3_)-2-keto-4-methylpentanoic acid in the cheese model: 383 μmol/kg curd.

At first sight, the conversion rates in the laboratory cheese model seemed to be relatively low because the total conversion rate of the l-leucine was only about 0.2% (Table 6). However, a comparison to the conversion rates of l-leucine metabolites determined in the original, unripened pilot-scale Gouda-type cheese had shown similar results (Table 10). Besides a similar concentration ratio of 3-methylbutanal vs 3-methyl-1-butanol (approximately 2:1) as well as the preference in the formation of 3-methylbutanoic acid, the total conversion rate of 0.4% was well within the range of the results obtained for the laboratory cheese model.

**Table 10:** Conversion Rates of l-Leucine into Three Metabolites Measured in Unripened Gouda Cheese

metabolite	conversion rate (%)
3-methylbutanal	0.11
3-methyl-1-butanol	0.05
3-methylbutanoic acid	0.27
total	0.43

To conclude, model studies using a CAMOLA approach by spiking cheese with (^13^C_6_)-l-leucine followed by a model experiment with (^2^H_3_)-2-keto-4-methylpentanoic acid confirmed l-leucine as the only source of the aroma compounds 3-methylbutanal, 3-methyl-1-butanol, and 3-methylbutanoic acid during ripening of Gouda cheese. The latter compound was preferentially formed by the direct degradation of 2-keto-4-methylpentanoic acid. Obviously, the lactobacilli strains preferentially generate the respective acids from amino acids in an Ehrlich mechanism. This is different from the degradation of amino acids by baker’s yeasts, which preferentially form the alcohol, i.e., 3-methylbutanol.^22^

## Supporting Information Available

Table S1: selected ions (MS/CI) and response factors used in the stable isotope dilution assays of 16 cheese aroma compounds; Table S2: concentrations of 16 aroma compounds at three ripening stages in cheese made from pasteurized milk (PM-G; batch 2); Table S3: concentrations of 16 aroma compounds at three ripening stages in cheese made from raw milk (RM-G cheese; batch 2) (PDF)

## AbbreviationsBBREVIATIONS

CAMOLA: carbon module labeling
DM: dry matter
PM-G: Gouda cheese made from pasteurized milk
RM-G: Gouda cheese made from raw milk
SAFE: solvent assisted flavor evaporation
SIDA: stable isotope dilution assay
