# Supplementary material for: Formation of Key Aroma Compounds During 30 Weeks of Ripening in Gouda-Type Cheese Produced from Pasteurized and Raw Milk
Source: J Agric Food Chem. 2024 May 3;72(19):11072–9. doi: 10.1021/acs.jafc.4c01814 (PMC11100003; doi:10.1021/acs.jafc.4c01814)
Supplement: Supplementary file 1 — jf4c01814_si_001.pdf [file jf4c01814_si_001.pdf]

Supplementary Information

Formation of Key Aroma Compounds during 30 Weeks of Ripening in Gouda-type Cheese Produced from Pasteurized and Raw Milk

[a]Philipp W. Duensing, [b]Jörg Hinrichs and [a]Peter Schieberle*

[a]former Chair for Food Chemistry; Faculty of Chemistry, Technical University of Munich; Lise-Meitner-Str. 34, D-85354 Freising, Germany

[b] Dep. Soft Matter Science and Dairy Technology, Institute of Food Science and Biotechnology, University of Hohenheim, Garbenstraße 21; D-70599 Stuttgart, Germany

* To whom correspondence should be addressed:

E-mail: Peter.Schieberle@ch.tum.de

Table S1. Selected ions (MS/CI) and response factors used in the stable isotope dilution assays of 16 Key Cheese Odorants

odorant	ion (*m/z*)	labeled standard (*m/z*)	ion (*m/z*)	RF [a]
butane-2,3-dione	87	($^{13}C_4$)-butane-2,3-dione	91	1.00
butanoic acid	89	($^{2}H_2$)-butanoic acid	91	0.87
δ-decalactone	171	($^{2}H_2$)-δ-decalactone	173	0.99
δ-dodecalactone	199	($^{2}H_2$)-δ-dodecalactone	201	1.01
acetic acid	61	($^{2}H_3$)-acetic acid	64	0.98
ethyl butanoate	117	($^{2}H_3$)-ethyl butanoate	120	0.95
ethyl hexanoate	145	($^{2}H_3$)-ethyl hexanoate	148	0.97
hexanoic acid	117	($^{2}H_3$)-hexanoic acid	120	0.81
2-methylpropanoic acid	89	($^{2}H_7$)-2-methylpropanoic acid	96	0.87
3-methylbutanal	69	($^{2}H_2$)-3-methylbutanal	71	0.98
3-methylbutanol	71	($^{2}H_2$)-3-methylbutanol	73	1.07
3-methylbutanoic acid	103	($^{2}H_2$)-3-methylbutanoic acid	105	0.87
pentanoic acid	103	($^{2}H_3$)-pentanoic acid	106	0.90
2-phenylacetic acid	137	($^{13}C_2$)-2-phenylacetic acid	139	0.86
2-phenylethanol	105	($^{13}C_2$)-2-phenylethanol	107	1.02

[a] MS response factor determined by analyzing defined mixtures of the analyte and the internal standard in MS/CI.

Table S2. Concentrations of 16 key odorants in three ripening stages in PM-G (batch 2)

odorant	concn (µg/kg DM) [a]		
	after 4 weeks	after 11 weeks	after 30 weeks
butanoic acid	11589	43033	155379
hexanoic acid	4302	7304	13756
pentanoic acid	170	256	553
ethyl butanoate	14	21	42
ethyl hexanoate	4	6	14
δ-dodecalactone	3035	5390	5391
δ-decalactone	2240	2509	2529
butane-2,3-dione	5396	5058	3651
3-methylbutanal	244	460	316
3-methyl-1-butanol	150	182	342
3-methylbutanoic acid	840	15326	27658
2-methylbutanoic acid	150	1246	2891
acetic acid	1917224	1946390	2090742
2-methylpropanoic acid	2715	10858	34273
2-phenylethanol	110	197	412
2-phenylacetic acid	419	1036	1588

[a] Concentrations determined in dry matter (DM). Mean value of at least three samples.

Table S3. Concentrations of 16 key odorants in three ripening stages in RM-G cheese (batch 2)

odorant	concn (µg/kg DM) [a]		
	after 4 weeks	after 11 weeks	after 30 weeks
butanoic acid	24327	83911	247537
hexanoic acid	10918	18739	43367
pentanoic acid	260	409	1072
ethyl butanoate	23	49	129
ethyl hexanoate	28	61	132
δ-dodecalactone	3017	4836	5136
δ-decalactone	2178	2502	2449
butane-2,3-dione	4595	2018	1019
3-methylbutanal	90	264	182
3-methyl-1-butanol	79	118	220
3-methylbutanoic acid	715	9361	16410
2-methylbutanoic acid	89	568	1792
acetic acid	1699945	2057828	2459792
2-methylpropanoic acid	2323	11576	33493
2-phenylethanol	84	131	332
2-phenylacetic acid	245	1192	2009

[a] Concentration calculated in dry matter (DM). Mean value of at least three samples.
